# Population mixing promotes arms race host–parasite coevolution

**DOI:** 10.1098/rspb.2014.2297

**Published:** 2015-01-07

**Authors:** Pedro Gómez, Ben Ashby, Angus Buckling

**Affiliations:** 1 Department of Biosciences, University of Exeter, Penryn Campus, Cornwall TR10 9FE, UK; 2 Department of Zoology, University of Oxford, Oxford OX1 3PS, UK

**Keywords:** antagonistic coevolution, arms race dynamic, bacteriophages, fluctuating selection dynamic, mixing, *Pseudomonas fluorescens*

## Abstract

The consequences of host–parasite coevolution are highly contingent on the qualitative coevolutionary dynamics: whether selection fluctuates (fluctuating selection dynamic; FSD), or is directional towards increasing infectivity/resistance (arms race dynamic; ARD). Both genetics and ecology can play an important role in determining whether coevolution follows FSD or ARD, but the ecological conditions under which FSD shifts to ARD, and vice versa, are not well understood. The degree of population mixing is thought to increase host exposure to parasites, hence selecting for greater resistance and infectivity ranges, and we hypothesize this promotes ARD. We tested this by coevolving bacteria and viruses in soil microcosms and found that population mixing shifted bacteria–virus coevolution from FSD to ARD. A simple theoretical model produced qualitatively similar results, showing that mechanisms that increase host exposure to parasites tend to push dynamics towards ARD. The shift from FSD to ARD with increased population mixing may help to explain variation in coevolutionary dynamics between different host–parasite systems, and more specifically the observed discrepancies between laboratory and field bacteria–virus coevolutionary studies.

## 1. Introduction

Host–parasite antagonistic coevolution, the reciprocal evolution of host defence and parasite counter-defence, can have important consequences for a wide range of ecological and evolutionary processes [1,2], including population dynamics [3], the maintenance of genetic diversity [4], the evolution of virulence [5] and the evolution of recombination [6] and mutation rates [7]. However, the impact of coevolution is contingent on the precise nature of the coevolutionary dynamics, a key feature being the extent to which coevolution follows an arms race dynamic (ARD) or a fluctuating selection dynamic (FSD) [8]. Under ARDs, host and parasites develop resistance and infectivity to an increasing range of genotypes through time (i.e. generalist strategies are increasingly favoured). Under FSDs, there are fluctuations in the frequency of genotypes with specialized resistance and infectivity (specialism FSDs) [8] or fluctuations in resistance and infectivity range (range FSDs) [9]. The extent of the implications of these different dynamics is unclear, but specialism FSD, for example, is associated with a fitness advantage of rare genotypes, and hence the maintenance of adaptive genetic diversity [10], selection for recombination [6,9] and host–parasite local adaptation [11]. It is, therefore, crucial to understand what determines the extent of ARD versus FSD.

Theoretical models suggest that the genetic basis of host–parasite specificity plays an important role in determining the extent of FSD versus ARD [6,9,12,13]. For example, if a specific infectivity allele is required to infect a host with a particular resistance allele (a ‘Matching Alleles Model’ (MAM) of infection genetics), then coevolution is most likely to follow specialism FSD [13]. At the other extreme of a continuum, a ‘Gene for Gene’ (GFG) model of infection genetics [14,15] allows the existence of single alleles that confer variable resistance and ranges, predisposing the system towards ARDs [13]. Consistent with a crucial role of genetics, different host–parasite systems tend to be associated with particular types of dynamics, for example, snails and trematodes [16] and daphnia and bacteria [17] are associated with specialism FSDs, and plants and fungi are most associated with ARDs [14,15,18].

The environment can also theoretically affect the extent of ARDs and FSDs [9,19,20]. Notably, ARDs can switch to either range or specialism FSDs under a GFG-type scenario if there are environment-dependent costs associated with elevated resistance and infectivity ranges [9,13,20,21]. While there are numerous examples in a range of systems suggesting that environmental conditions can alter coevolutionary dynamics [22–26], evidence to date for the environment unambiguously shifting dynamics between FSD and ARD is limited to microcosm studies involving bacteria and their obligate killing viruses (lytic bacteriophages). Specifically, the same combination of bacteria and virus (the soil bacterium *Pseudomonas fluorescens* SBW25 and the virus SBW25φ2; [27]) has been shown *in vitro* to undergo largely ARDs in nutrient-rich media, with dynamics shifting towards range FSDs in lower-nutrient media [20]. Moreover, when cultured in soil-based growing media (compost), the organisms undergo specialism FSDs [28].

In this study, we investigate how a key environmental variable, the degree of population mixing, affects the extent of ARD versus FSD. Species and populations are typically spatially structured, and the extent of gene flow between populations can alter coevolutionary dynamics and result in complex patterns of local adaptation and maladaptation to other species across landscapes [1,29–32]. The key consequence of population mixing in the context of ARDs versus FSDs is likely to be the higher encounter rates between host and parasites [33–35]. Such increased encounter rates may increase the selective advantage of evolving broad resistance ranges, resulting in coevolution shifting away from FSDs towards ARDs. We tested this hypothesis by developing a simple theoretical model and carrying out experimental bacteria–virus coevolution in compost microcosms that are mixed to varying degrees. We also carried out nutrient addition experiments to help identify if mixing *per se* or changes in population densities were responsible for our observed empirical results.

## 2. Material and methods

### (a) Mixing experiment

*Pseudomonas fluorescens* SBW25 strain marked for resistance to gentamicin [28] was grown overnight at 28°C in King's media B (KB) in an orbital shaker (180 r.p.m.) and then centrifuged for 10 min at 3500 r.p.m. to produce a bacterial pellet, which was resuspended in M9 buffer to a final concentration of 10^8^ colony forming units (CFUs ml^−1^). Twenty-four polypropylene trays (4 treatments × 6 replicates per treatment), containing 100 g of twice-autoclaved compost (John Innes no. 2) soil (soil microcosm) were inoculated with a natural-soil microbial community from a soil wash (20 g of soil × 100 ml^−1^ M9 buffer). The next day, 12 microcosms were inoculated with 5 ml of M9 salt solution containing a suspension (10^6^ plaque forming units (PFUs)) of the virulent bacteriophage SBW25φ2 initiated from a single clone. Five millilitres of M9 salt solution were added to the other 12 microcosms. Then, 5 ml of the *P. fluorescens* suspension (10^8^ CFUs) were inoculated into all microcosms a day later. Soil microcosms were placed in an environmental chamber at 26°C and 80% relative humidity. Half of the microcosms from each treatment were mixed using a sterile spatula every day [28].

We established a third treatment that resulted in more extensive mixing (the soil–water treatment). We inoculated 12 × 30 ml glass bottles containing 6 ml of soil–water (3 g of soil × 6 ml^−1^ sterile water) with *P. fluorescens* SBW25 (10^8^ CFUs), and half with phage SBW25φ2 (10^6^ PFUs). Populations were propagated at 28°C in an orbital shaker at 200 r.p.m. Fifty per cent of each culture was transferred to fresh soil–water approximately every 5 days, for three transfers; preliminary work showed that populations started to decline in density after 5 days, as is commonly observed in batch culture [36].

### (b) Nutrient availability experiment

Twenty-four soil microcosms (4 treatments × 6 replicates) were inoculated with *P. fluorescens* SBW25, with half additionally inoculated with phage SBW25φ2, as described above. Immediately prior to inoculating with bacteria and phage, 12 replicates (half with phages) were inoculated with KB (5 ml), and 12 with sterile water, and mixed using a sterile spatula.

### (c) Sample collection

At each time point, soil samples (2 g) were collected using a sterile spatula and mixed with 10 ml sterile M9 buffer and glass beads, and then vortexed for 1 min. The resultant soil washes were diluted and plated onto KB agar supplemented with gentamicin (15 µg ml^−1^ KB) and incubated for 2 days at 28°C to determine CFUs per gram of soil. To isolate phages, a sample of each soil wash was vortexed with 10% chloroform and centrifuged at 13 000 r.p.m. The phage supernatant was plated onto exponentially growing ancestral bacteria in 0.6% KB agar to enumerate PFUs. From each replicate population and time point sampled, 12 bacterial clones and a phage suspension were stored at −20°C in glycerol solution (20%). Note that no culturable bacteria were detected that could grow on KB supplemented with gentamicin, nor could they be infected by phage SBW25φ2. Moreover, we did not find any phages in the soil wash that were able to infect *P. fluorescens* SBW25 [28].

### (d) Resistance and infectivity assays

The 12 *P. fluorescens* clones from each population were assayed for resistance by streaking the bacteria against a line of phage (50 µl) on KB agar; growth inhibition indicated sensitivity [27,37].

### (e) Measurement of coevolution

Bacteria clones isolated from day 9 were assayed for resistance (proportion of resistant colonies) to phages from ancestral (day 0), contemporary (day 9) and future (day 14) populations from within the same communities. Likewise, phages from day 9 were assayed against bacteria from days 0, 9 and 14. Bacteria and phage from each time point were all also tested against their contemporary phage and bacteria populations, as well as the ancestral clones.

### (f) Statistical analyses

Bacteria and phage densities, and bacterial resistance to contemporary and ancestral phages, were averaged through time for each replicate. The transformed data (log_10_ for density and square-root (arcsine) for proportion resistant bacteria) were analysed as General Linear Models (GLM), fitting treatments and their interactions as appropriate. Coevolutionary dynamics were determined by analysing the proportion of resistant bacteria as GLM for each separate treatment, fitting replicates (1–6) and time as both linear and quadratic terms. All analyses were carried out using JMP (v. 9) software. Note that our test of specialism FSD, resistance or infectivity consistently peaking for contemporary interactions, is highly conservative, as fluctuations may not be parallel between replicates. However, distinguishing non-parallel fluctuations in phenotypic traits from random error is problematic [38,39]. We do not focus on range FSD in this study (which would require measurement of infectivity and resistance ranges over multiple time points [20]) given that bacteria and phage experience specialism FSD, and not range FSD, in unmixed soil microcosms [28].

### (g) Model description

Simulations were conducted using a modified version of the model proposed by Ashby *et al.* [40]. Space was represented by a two-dimensional square lattice of side length *N*, where each site was either empty or was occupied by a single sessile host (bacterium). Infected hosts were killed after *τ* time steps and released *β* new parasites into the environment. Parasites (phages) were allowed to spread through the environment with diffusion constant *D* and infected hosts based on their local concentration (*P*), specificity to the host at the same site (*Q*), fitness costs associated with broader ranges (*c*_P_) and fixed rates of adsorption (*α*) and decay (*δ*) (see [40] for full description of the simulation rules). Hosts were either randomly redistributed in space at the end of each time step, analogous to the mixed experiment, or were left unmixed.

The aim of our modelling approach was to determine qualitative outcomes of population mixing and other variables, rather than specifically modelling the details of our bacteria–phage system (which we do not yet know enough about). However, it was of course important to capture the qualitative coevolutionary dynamics of the bacteria–phage system, namely, that both ARDs and specialism FSDs can occur to some extent. To this end, host–parasite specificity (*Q*) was based on interactions at three biallelic loci, two of which affected the range of genotypes that could be resisted/infected ('symmetric gene-for-gene’, SGFG). Note that we do not use a normal GFG model because this assumes an implicit genetic asymmetry in favour of parasites, which is a model specifically associated with certain plant–pathogen interactions [15] (and does not seem to be case across a wide range of bacteria–phage systems [41]), hence the SGFG appears to be more general. The remaining locus determined how specific the parasite was to the host (‘matching-alleles’, MA). This approach, which is similar to other two-step models of specificity [42,43], allows both ARDs (SGFG loci) and FSDs (MA locus) to occur. Genotypes for both populations are of the form XY/Z, where X and Y are SGFG loci and Z is the MA locus. For hosts, the presence (1) of a resistance allele at a locus where the parasite does not have an infectivity allele (0) results in a reduction in infectivity (*Q*) by a factor of 0 < *σ* < 1. For parasites, the presence (1) of an infectivity allele at a locus where the host does not have a resistance allele (0) results in an increase in infectivity by a factor of 1/*σ*, up to a maximum of *Q* = 1. The MA locus may contain either an ‘A’ or a ‘B’ allele. Mismatches at the MA locus result in a reduction in infectivity by a factor of 0 < *ρ* < 1. Electronic supplementary material, table S1 shows the full set of genotype by genotype interactions possible in the model.

### (h) Model analysis

Two hundred and fifty simulations were conducted for low and high values of the adsorption parameter *α*, which modified the force of infection experienced by the host. The peak resistance and infectivity ranges of hosts and parasites (i.e. the maximum frequency of resistance and infectivity alleles) were measured for each simulation where coexistence was observed for 10 000 time steps, as was the variance in the frequency of alleles at each locus. The temporal variance at the MA locus (*V*_MA_) was then compared with the temporal variance at all loci (*V*_ALL_), so that the relative importance of FSDs to ARDs could be ascertained. If *V* = *V*_MA_/*V*_ALL_ was greater than 0.5 in a given simulation, then FSD were more common than ARD, whereas *V* < 0.5 indicated that ARD were more common. We fix the remaining model parameters to the following: *β* = 100, *δ* = 0.5, *ɛ*_H_ = 0.002, *ɛ*_P_ = 0.02, *ρ* = 0.25, *η*_H_ = 0.15, *η*_P_ = 0.2, *μ* = 0.1, *σ* = 0.8, *τ* = 1, *N* = 50, *T* = 1 (*ρ* and *σ* described in the electronic supplementary material; other parameters fully described in Ashby *et al.* [40]).

## 3. Results

### (a) Experiments

**Figure 1.**
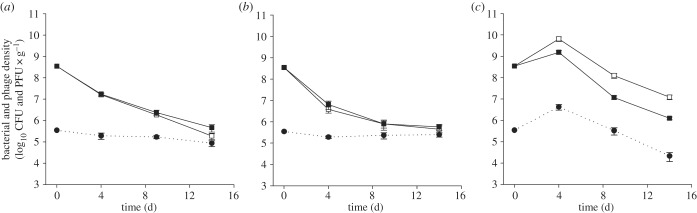
Population dynamics of bacteria and phage under different mixing regimes. Mean densities (±s.e.m.) of *P. fluorescens* SBW25 (solid lines; CFUs g^−1^ soil) and phage SBW25φ2 (dotted lines; PFUs g^−1^ soil) populations under no (*a*), daily (*b*) and soil–water (*c*) mixing treatments. Bacteria population densities were determined in the absence (unfilled squares) and presence (filled squares) of phages.

To investigate the role of population mixing on coevolutionary dynamics between *P. fluorescens* SBW25 and φ2, we used three treatments: no mixing; daily mixing and continual mixing, by shaking soil in water (soil–water treatment). We also included phage-free control populations under all of the mixing regimes. After 4, 9 and 14 days, we sampled microcosms to assess population densities and bacterial resistance to phage populations both within and across time points of coevolving communities. The mixing treatments increased the mean density of bacteria (figure 1; *F*_2,30_ = 48.40, *p* < 0.001) and phages (figure 1; *F*_2,15_ = 4.72, *p* = 0.026). This increase in bacterial density with increasing mixing in soil presumably resulted from increased access to nutrients and space. Phages caused a mean reduction in bacterial densities (figure 1; *F*_1,30_ = 2.59, *p* < 0.012).

**Figure 2.**
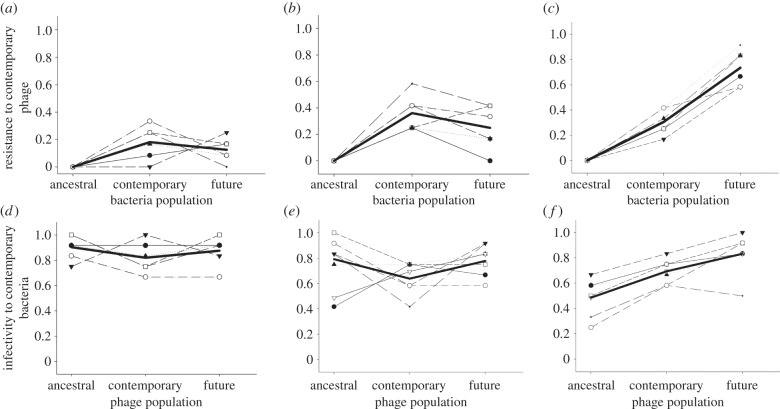
Coevolutionary dynamics of bacteria and phages under different mixing regimes. (*a–c*) The proportion of *P. fluorescens* clones isolated from days 0, 9 and 14 that are resistant to phages populations isolated from day 9 under no (*a*), daily (*b*) and soil–water (*c*) mixing treatments. (*d–f*) The proportion of *P. fluorescens* clones isolated from day 9 that are susceptible (i.e. phage infectivity) to phage populations from days 0, 9 and 14 under no (*d*), daily (*e*) and continual (*f*) mixing treatments. Individual thin lines show the six separate replicates in each treatment; the bold line shows the average for all replicates.

Determining the qualitative coevolutionary dynamic (i.e. whether predominantly ARD or specialism FSD) requires measurement of bacterial resistance and phage infectivity to past, contemporary and future phage and bacteria populations, respectively [8,39,44,45]. A tendency for resistance and infectivity of future bacteria and phage, respectively, to be greater than past bacteria and phage is indicative of ARD, while higher resistance or infectivity of contemporary bacteria and phage compared with both past and future populations would provide unambiguous evidence of FSD [8]. We therefore measured the resistance of contemporary (day 9) bacteria to contemporary, past (day 4) and future (day 14) phage populations, and the infectivity of contemporary phage to contemporary, past and future bacterial populations within each replicate. Under both no and daily mixing conditions, bacteria resistance peaked against contemporary phages (figure 2*a,b*; quadratic term: *F*_1,10_ = 6.04, *p* < 0.03; *F*_1,10_ = 26.87, *p* < 0.001, respectively), although infectivity of phage did not significantly differ through time (figure 2*d,e*; *p* > 0.1 in both cases). By contrast, the soil–water treatment resulted in an increase in both resistance (figure 2*c*; linear term: *F*_1,10_ = 287.99, *p* < 0.001) and infectivity (figure 2*f*; linear term: *F*_1,10_ = 35.34, *p* < 0.001) through time; quadratic terms were non-significant (*p* > 0.2) in both cases. These data demonstrate that coevolutionary dynamics were consistent with specialism FSD under no and intermediate mixing treatments and ARD under the soil–water mixing regime. Note that there was no detectable resistance in populations of bacteria evolved in the absence of phages.

**Figure 3.**
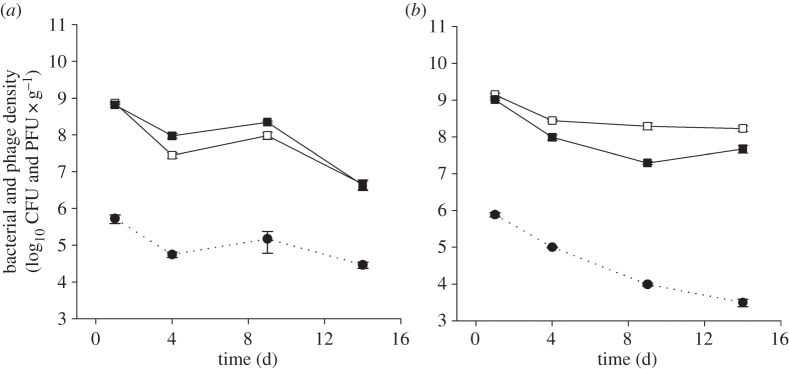
Population dynamics of bacteria and phage with the addition of nutrients. Mean densities (±s.e.m.) of *P. fluorescens* SBW25 (solid lines; CFUs g^−1^ soil) and phage SBW25φ2 (dashed lines; PFUs g^−1^ soil) populations with the addition of water (*a*) and KB media (*b*). Bacteria population densities were determined in the absence (unfilled squares) and presence (filled squares) of phages.

**Figure 4.**
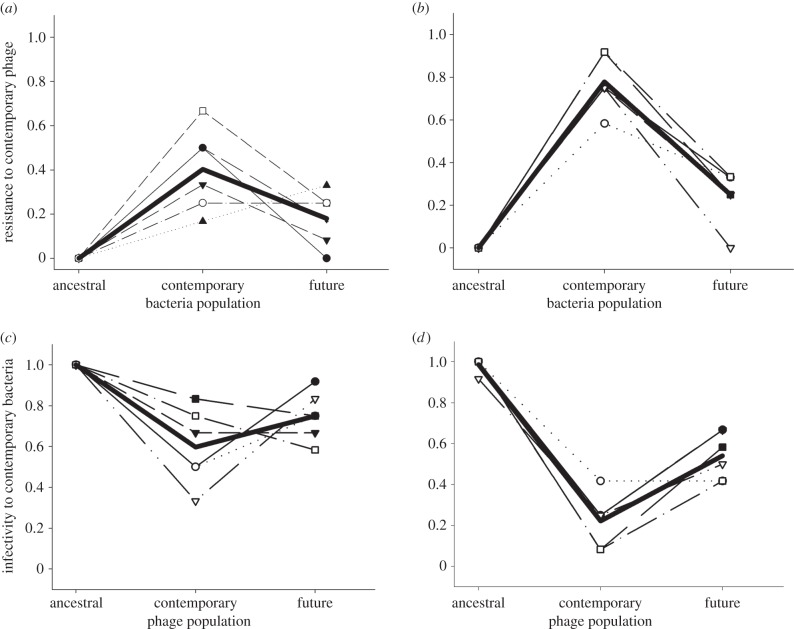
Coevolutionary dynamics of bacteria and phages with the addition of water or nutrients. (*a*,*b*) The proportion of *P. fluorescens* clones isolated from days 0, 9 and 14 that are resistant to phage populations isolated from day 9 with the addition of water (*a*) and KB media (*b*). (*c*,*d*) The proportion of *P. fluorescens* clones isolated from day 9 that are susceptible (i.e. phage infectivity) to phage populations from days 0, 9 and 14 with the addition of water (*c*) and KB media (*d*). Individual thin lines show the six separate replicates in each treatment; the bold line shows the average for all replicates.

We next investigated the role of nutrient availability on coevolutionary dynamics in soil. Previous studies showing bacteria–phage ARDs *in vitro* were primarily carried out in high-nutrient KB [27,39], so we increased nutrients simply by adding KB to soil. While the addition of KB media increased the densities of bacteria (figure 3; *F*_1,20_ = 32.98, *p* < 0.001), phage densities were reduced (figure 3; *F*_1,10_ = 23.39, *p* < 0.001). In contrast to the soil–water treatment, nutrient addition did not affect the qualitative coevolutionary dynamics. In both treatments, bacterial resistance peaked against contemporary phages (figure 4*a,b*; quadratic terms: *F*_1,10_ = 27.24, *p* < 0.001; *F*_1,10_ = 110.71, *p* < 0.001, respectively) and phage infectivity was lowest against contemporary bacteria (figure 4*c,d*; quadratic terms: *F*_1,10_ = 28.15, *p* = 0.003; *F*_1,10_ = 109.11, *p* < 0.001, respectively). Therefore, FSD was maintained despite the addition of nutrients to the soil.

While nutrient addition and mixing resulted in different coevolutionary dynamics, both manipulations caused a significant increase in mean resistance to phages (figures 2 and 4; electronic supplementary material, figures S1 and S2; mixing: *F*_2,30_ = 48.40, *p* < 0.001; nutrient addition: *F*_1,20_ = 14.54, *p* < 0.001).

### (b) Model results

**Figure 5.**
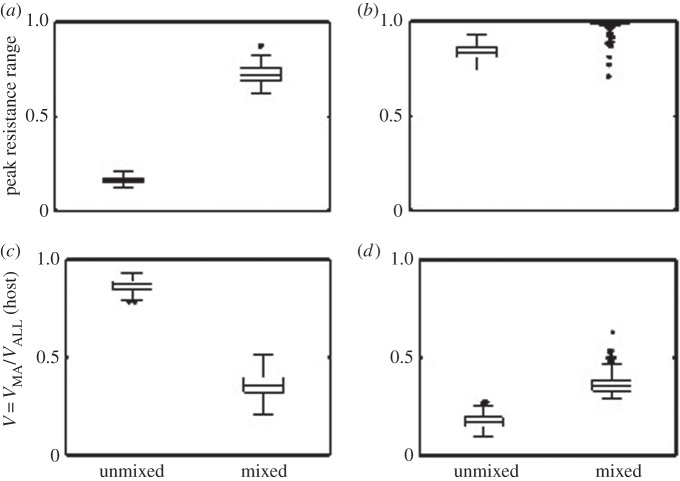
Modelling simulation results. (*a*,*b*) Peak resistance range (i.e. the proportion of loci that contained a resistance allele) and (*c*,*d*) the relative variance at the MA locus in unmixed and mixed environments for the host (see §2 for full model description). Values of *V* > 0.5 indicate FSDs were more important than ARDs, and vice versa for *V* < 0.5. (*a*,*c*) Data for a low-adsorption rate (*α* = 0.05); (*b*,*d*) Data for a high-adsorption rate (*α* = 0.1). Low-adsorption rates produced contrasting coevolutionary dynamics in unmixed and mixed environments: ARD occurred in mixed environments, whereas FSD occurred in unmixed environments. Higher adsorption rates increased the force of infection (and hence selection) on the host, leading to ARD in both environments.

We set up simulations of host–parasite coevolution where the genetics of infectivity/resistance were simultaneously determined by two types of loci. One type of loci governed the range of genotypes that could be resisted or infected, while the other controlled specialization on subsets of genotypes. Greater variance in the first type of loci is indicative of ARD, whereas greater variance in the second type of loci is indicative of FSD. Under conditions where the probability of infection was relatively low, mixing shifted host dynamics from FSD towards ARD (figure 5), as determined by the proportion of total variance that occurred at the locus that affected only specialisation. Crucially, we did not find any conditions where the reverse was true, suggesting that ARD is more probably to be associated with reduced spatial structure. However, increasing the probability of infection through greater adsorption rates (the probability of infection for a given host–parasite encounter rate) or by increasing the encounter rate (e.g. greater burst size, lower decay rate) resulted in ARD in both mixed and unmixed environments. In contrast to the host, the parasite was always under selection to accumulate infectivity alleles (ARD), but mixed environments tended to favour greater range expansion (electronic supplementary material, figure S3).

## 4. Discussion

In this study, we investigated how population mixing affects coevolutionary dynamics between bacteria and phage in soil. We found that mixing shifted coevolution from specialism FSDs, with bacteria most resistant to contemporary compared with past or future phage populations, to ARDs, where bacteria resistance and phage infectivity ranges increased through time. Our model suggests a very general explanation for this: mixing exposes hosts to more parasites, selecting for wider resistance ranges. If parasites require broad ranges to infect highly resistant hosts, as is the case in this [46] and other bacteria–virus [47] and plant–pathogen [15] systems, there will be reciprocal selection for parasite generalism, resulting in an ARD. Crucially, modifying any variable in our model that increases host exposure to parasites, such as greater parasite fecundity or a reducing the rate of decay outside the host, has a qualitatively similar effect on coevolutionary dynamics.

In contrast to mixing soil, nutrient addition did not cause a shift from FSD to ARD. Both mixing and nutrient addition increased bacterial densities to a similar extent, but whereas mixing also increased phage densities, nutrient addition caused a reduction. As a result, mixing presumably caused a greater increase in encounter rates than did nutrient addition. Despite nutrient addition not affecting the qualitative coevolutionary dynamics, mean resistance to phages was increased. This is likely to be because the physiological costs of phage resistance are reduced with increased nutrient availability (over and above any demographic effects) in this system [37].

Shaking soil in water could of course have many other effects on bacteria and phage interactions over and above encounter rates, as is the case for any experimental manipulation of population structure. However, we believe we can rule out these additional effects as likely explanations for our results. First, while the soil–water regime is likely to have released more nutrients, and these nutrients were replenished by transferring bacteria to new soil–water (unlike the other treatments), nutrient availability is an unlikely explanation given the results of the nutrient addition experiment. Second, *in vitro* work has shown that the ARD switches towards FSD through time [39], and it is possible that different rates of transition could explain our results. However, if anything, we would expect this transition from ARD to FSD to occur faster, not slower, in the soil–water treatment because of larger population sizes and hence more rapid evolution. Third, *P. fluorescens* can diversify into resource specialists [48,49] with intrinsic differences in phage resistance [50] in structured environments; however, this diversity does not influence qualitative coevolutionary dynamics *in vitro* [34,51]. We therefore suggest that population mixing directly affects coevolutionary dynamics by altering encounter rates between bacteria and phages.

While our model shows that mixing can shift FSD towards ARD for hosts, we do not observe any FSD for parasites, with selection always favouring the accumulation of infectivity alleles. This probably reflects that both the genetics and ecology of the interaction are more complicated than our model, but there is still consistency between the simulation results and the experimental data in that the signature of FSD is stronger for the host than the parasite. This finding may reflect different strengths of selection acting on the host and parasite: if encounter rates are relatively low, there will be extremely strong selection acting on parasites to maximize their chance of infection, hence favouring broader host ranges.

Although there is a large body of theoretical and empirical work on how population mixing can affect the causes and consequences of coevolution, we have shown that population mixing is likely to be an important determinant of whether coevolution follows an ARD or FSD, and hence the impact of coevolution of population dynamics and the resultant evolution of other traits [1–7]. Soil disturbances, both natural and agricultural, may therefore dramatically alter bacteria–phage interactions, and microbial community structure as a whole [52]. Finally, the results may help to explain some of the discrepancies between field and laboratory studies of bacteria–virus coevolution; the former is typically associated with FSDs [53,54] and the latter with ARDs [41,55]. Microbes are commonly attached to particles [56,57], hence natural environments are likely to be more spatially structured than laboratory media. Whether the results hold for non-bacteria–virus systems is currently unclear, although the genetic bases of coevolutionary interactions are typically very complex and may allow both ARD and FSD [58], as captured in our model. Moreover, the lack of reported shifts between ARDs and FSDs in other systems may well reflect the difficulty of unambiguously determining coevolutionary dynamics [8,12,45] in the absence of detailed time-course data rather than an absence of an environmental effect.

## Supplementary Material

ESM
