# Supplementary material for: Population mixing promotes arms race host–parasite coevolution
Source: Proc Biol Sci. 2015 Jan 7;282(1798):20142297. doi: 10.1098/rspb.2014.2297 (PMC4262181; doi:10.1098/rspb.2014.2297)

Supplementary material (on Manuscript ID RSPB-2014-2297)

Table S1. Host-parasite specificity based on interactions at two types of biallelic loci (SGFG and MA).

Infectivity, Q		Parasite genotype							
		00/A	00/B	01/A	01/B	10/A	10/B	11/A	11/B
Host genotype	00/A	**σ^2**	$\sigma^2\rho$	**σ**	$\sigma\rho$	**σ**	$\sigma\rho$	**1**	ρ
	00/B	$\sigma^2\rho$	**σ^2**	$\sigma\rho$	**σ**	$\sigma\rho$	**σ**	ρ	**1**
	01/A	**σ^3**	$\sigma^3\rho$	**σ^2**	$\sigma^2\rho$	**σ^2**	$\sigma^2\rho$	**σ**	$\sigma\rho$
	01/B	$\sigma^3\rho$	**σ^3**	$\sigma^2\rho$	**σ^2**	$\sigma^2\rho$	**σ^2**	$\sigma\rho$	**σ**
	10/A	**σ^3**	$\sigma^3\rho$	**σ^2**	$\sigma^2\rho$	**σ^2**	$\sigma^2\rho$	**σ**	$\sigma\rho$
	10/B	$\sigma^3\rho$	**σ^3**	$\sigma^2\rho$	**σ^2**	$\sigma^2\rho$	**σ^2**	$\sigma\rho$	**σ**
	11/A	**σ^4**	$\sigma^4\rho$	**σ^3**	$\sigma^3\rho$	**σ^3**	$\sigma^3\rho$	**σ^2**	$\sigma^2\rho$
	11/B	$\sigma^4\rho$	**σ^4**	$\sigma^3\rho$	**σ^3**	$\sigma^3\rho$	**σ^3**	$\sigma^2\rho$	**σ^2**

The presence (1) and absence (0) of resistance and infectivity alleles at SGFG loci are shown on the left side of each genotype. For hosts, the presence of a resistance allele at a locus where the parasite does not have an infectivity allele results in a reduction in infectivity (Q) by a factor of $0<\sigma<1$. For parasites, the presence of an infectivity allele at a locus where the host doesn't have a resistance allele results in an increase in infectivity by a factor of $1/\sigma$, up to a maximum of Q=1. The MA locus (right hand side of

each genotype) may contain either an 'A' or a 'B' allele. Bold text indicates when parasites match the MA locus of a host; mismatches result in a reduction in infectivity by a factor of 0<ρ<1.

Figure S1. Resistance of bacteria under different mixing regimes. Mean proportion resistant (±SEM) *P. fluorescens* SBW25 against ancestral (☐) and contemporary (■) phage under no (A), daily (B) and soil-water (C) mixing treatments.

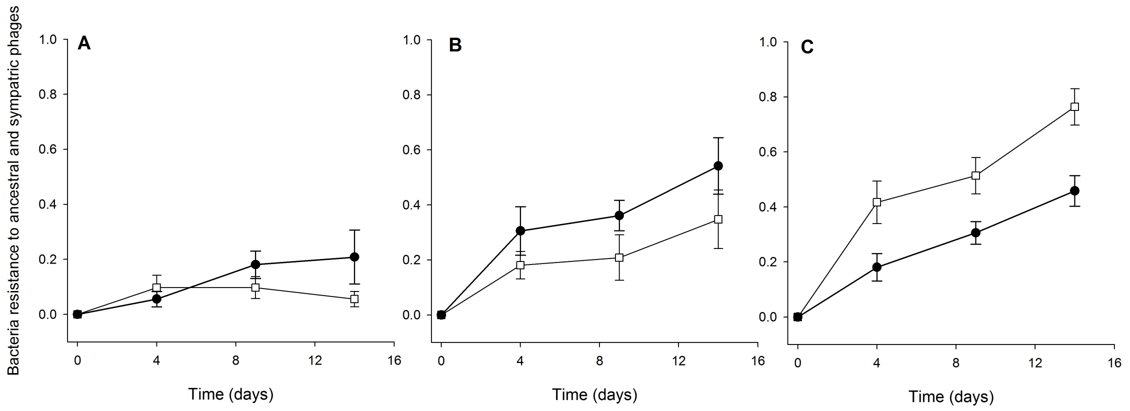

Figure S2. Resistance of bacteria with the addition of nutrients. Mean proportion resistant (±SEM) *P. fluorescens* SBW25 against ancestral (☐) and contemporary (■) phage with the addition of water (A) and KB media (B).

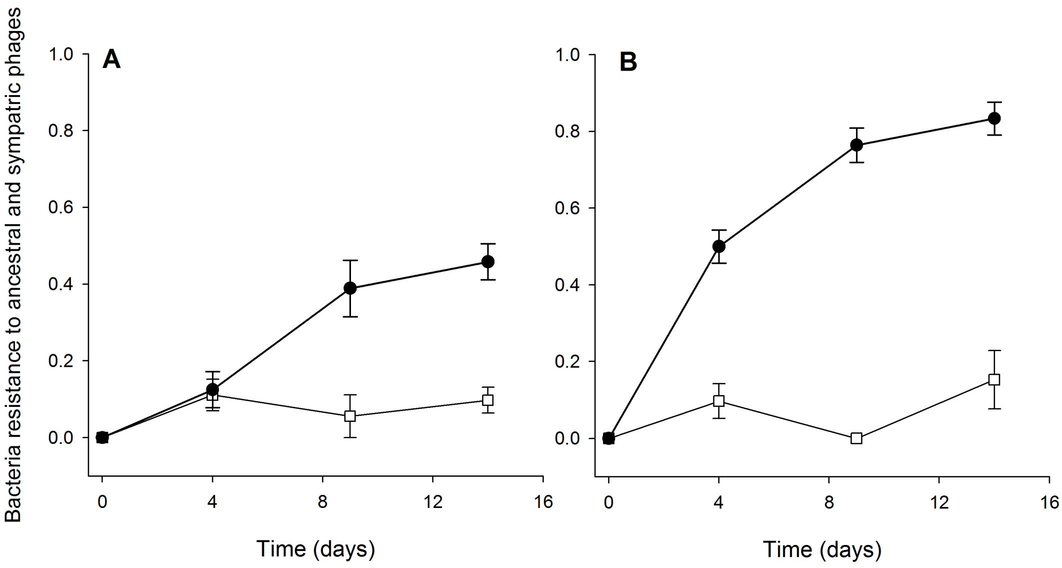

Figure S3. Modelling simulation results. (A-B) show peak infectivity range (i.e. the proportion of loci that contained an infectivity allele) and (C-D) the relative variance at the MA locus in unmixed and mixed environments for the parasite (see materials and methods for full model description). Values of V>0.5 indicate fluctuating selection dynamics (FSD) were more important than arms race dynamics (ARD), and vice versa for V<0.5. (A) and (C) show data for a low adsorption rate (α=0.05); (B) and (D) show data for a high adsorption rate (α=0.1). The parasite was always under selection to accumulate infectivity alleles (ARD), but mixed environments tended to favour greater range expansion. Higher adsorption rates led to stronger ARD in unmixed environments, due to host range expansion (Fig 5).

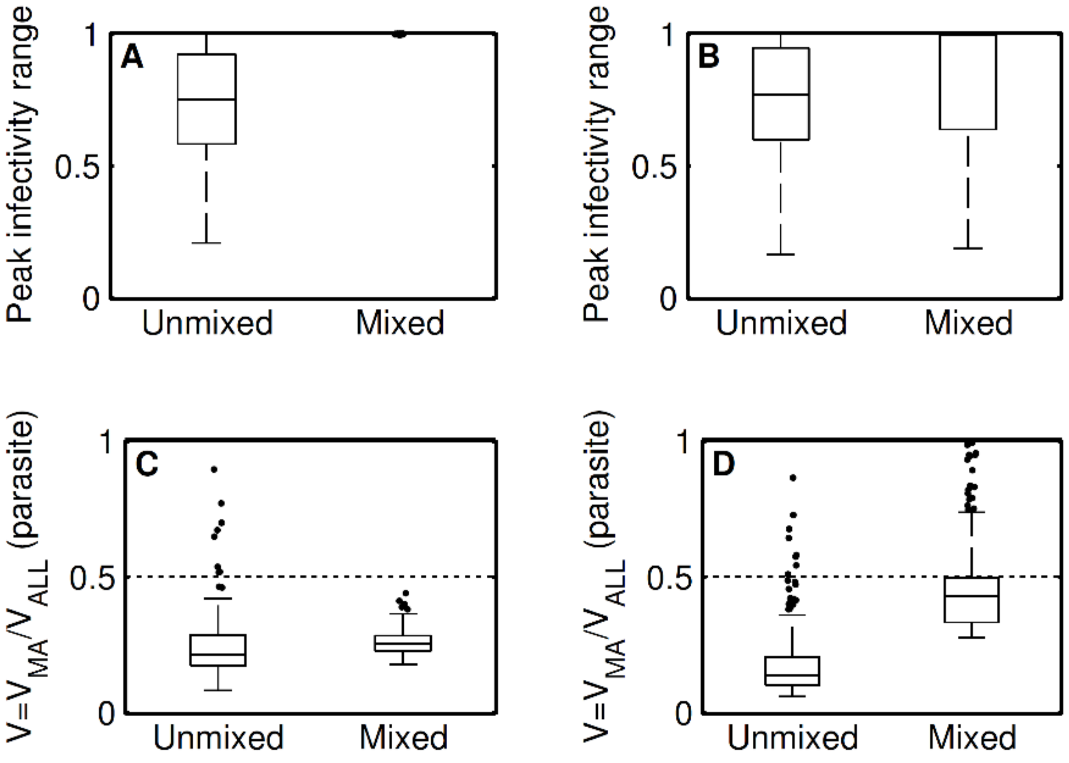

Supplement: ESM [file rspb20142297supp1.pdf]
